# Safety and efficacy of vismodegib in patients aged ≥65 years with advanced basal cell carcinoma

**DOI:** 10.18632/oncotarget.12660

**Published:** 2016-10-14

**Authors:** Anne Lynn S. Chang, Karl D. Lewis, Sarah T. Arron, Michael R. Migden, James A. Solomon, Simon Yoo, Bann-Mo Day, Edward F. McKenna, Aleksandar Sekulic

**Affiliations:** ^1^ Stanford University, Redwood City, CA, USA; ^2^ University of Colorado, Aurora, CO, USA; ^3^ University of California, San Francisco, San Francisco, CA, USA; ^4^ The University of Texas MD Anderson Cancer Center, Houston, TX, USA; ^5^ Ameriderm Research, Ormond Beach, FL, USA; ^6^ University of Central Florida, Orlando, FL, USA; ^7^ University of Illinois, Urbana, IL, USA; ^8^ Northwestern University, Chicago, IL, USA; ^9^ Genentech, Inc., South San Francisco, CA, USA; ^10^ Mayo Clinic, Scottsdale, AZ, USA

**Keywords:** basal cell carcinoma, vismodegib, age, Hedgehog pathway inhibitor, locally advanced basal cell carcinoma

## Abstract

Because many patients with unresectable basal cell carcinoma (BCC) are aged ≥65 years, this study explores the efficacy and safety of vismodegib in these patients with locally advanced (la) or metastatic (m) basal cell carcinoma (BCC) in the ERIVANCE BCC trial and the expanded access study (EAS).We compared patients aged ≥65 years to patients aged <65 years taking vismodegib 150 mg/day, using descriptive statistics for response and safety. Patients aged ≥65 years (laBCC/mBCC) were enrolled in ERIVANCE BCC (33/14) and EAS (27/26). Investigator-assessed best overall response rate in patients ≥65 and <65 years was 46.7%/35.7% and 72.7%/52.6% (laBCC/mBCC), respectively, in ERIVANCE BCC and 45.8%/33.3% and 46.9%/28.6%, respectively, in EAS. These differences were not clinically meaningful. Safety was similar in both groups, although those aged ≥65 years had a higher percentage of grade 3-5 adverse events than those aged <65 years. Vismodegib demonstrated similar clinical activity and adverse events regardless of age.

## INTRODUCTION

Therapeutic options are limited for patients with advanced basal cell carcinoma (aBCC), including locally advanced (laBCC) or metastatic (mBCC) disease. Aberrant activation of the Hedgehog pathway has been identified as a key driver in the pathogenesis of BCC, and was first described in patients with basal cell carcinoma nevus syndrome (BCCNS) [1, 2]. Vismodegib, the first Hedgehog pathway inhibitor to be approved by the US Food and Drug Administration (FDA), is indicated for patients who have aBCC that has recurred after surgery or who are not candidates for surgery and radiation [3]. In the pivotal aBCC study (ERIVANCE BCC), vismodegib demonstrated an objective response rate of 43% in patients with laBCC and 30% in patients with mBCC by independent review [3].

Patients aged ≥65 years have had longer exposure to ultraviolet light and may have more difficulty identifying new or changing skin lesions because of the presence of other age-related changes. For example, patients in this age group are more likely than younger patients to have declining eyesight or age-related skin changes such as actinic lentigo, seborrheic keratosis, or actinic keratosis [4]. Therefore, older patients are more likely to present with multiple lesions or with more advanced or neglected lesions than younger patients [5, 6]. Furthermore, patients aged ≥65 years may be more vulnerable to adverse events (AEs) from anticancer treatments because of comorbid medical conditions, age-related reductions in organ or cognitive function, and reduced physiologic reserves, resulting in a shift in the overall benefit-risk ratio of treatment [7].

Here, we present the efficacy and safety of vismodegib in patients aged ≥65 years with aBCC compared with younger patients who were all enrolled in the ERIVANCE BCC pivotal trial [3] and the US expanded access study (EAS) [8].

## RESULTS

### Patients

The ERIVANCE BCC trial enrolled 104 patients, including 71 (68%) and 33 (32%) with laBCC and with mBCC, respectively; 33 (46%) patients with laBCC and 14 (42%) patients with mBCC were aged ≥65 years. The EAS study enrolled 119 patients, of whom 62 (53%) and 57 (47%) had laBCC and mBCC, respectively; 27 (43%) patients with laBCC and 26 (46%) patients with mBCC were aged ≥65 years. Apart from age, baseline demographic and disease characteristics were generally similar between patients aged ≥65 years and those aged <65 years (Table 1). Concomitant medical conditions (e.g., hypertension, hyperlipidemia) were more frequent in patients aged ≥65 years than in younger patients. Basal cell carcinoma nevus syndrome, headache, depression, insomnia, and seasonal allergies were more common in younger patients.

**Table 1:** Patient demographics and baseline disease characteristics

	ERIVANCE BCC (*N* = 104)				EAS (*N* = 119)			
	laBCC		mBCC		laBCC		mBCC	
	≥65 years (*n* = 33)	<65 years (*n* = 38)	≥65 years (*n* = 14)	<65 years (*n* = 19)	≥65 years (*n* = 27)	<65 years (*n* = 35)	≥65 years (*n* = 26)	<65 years (*n* = 31)
Median age, years (range)	75.0 (65–101)	50.5 (21–62)	71.5 (66–92)	53 (38–64)	77 (67–92)	53 (26–63)	71.5 (65–100)	55 (24–63)
Female, *n* (%)	15 (45.5)	17 (44.7)	4 (28.6)	5 (26.3)	4 (14.8)	15 (42.9)	4 (15.4)	8 (25.8)
White, *n* (%)	33(100.0)	38 (100.0)	14 (100.0)	19 (100.0)	27 (100.0)	33 (94.3)	25 (96.2)	31 (100.0)
ECOG PS, *n* (%)								
0	22(66.7)	29 (76.3)	5 (53.7)	8 (42.1)	12 (44.4)	27 (77.1)	14 (53.8)	16 (51.6)
1	7(21.2)	8 (21.1)	9 (64.3)	10 (52.6)	12 (44.4)	7 (20.0)	10 (38.5)	12 (38.7)
2	4(12.1)	1 (2.6)	0	1 (5.3)	3 (11.1)	1 (2.9)	2 (7.7)	3 (9.7)
No. of target lesions, *n* (%)								
1	26(78.8)	22 (57.9)	4 (28.6)	5 (26.3)	16 (59.3)	18 (51.4)	12 (46.2)	12 (38.7)
2	5 (15.2)	7 (18.4)	2 (14.3)	2 (10.5)	6 (22.2)	7 (20.0)	4 (15.4)	6 (19.4)
≥3	2 (6.1)	9 (23.7)	8 (57.2)	12 (63.3)	5 (18.5)	10 (28.5)	10 (38.5)	13 (42.0)
Prior treatment, *n* (%)								
Surgery	27 (81.8)	35 (92.1)	14 (100.0)	18 (94.7)	25 (92.6)	32 (91.4)	25 (96.2)	29 (93.5)
Radiotherapy	13 (39.4)	9 (23.7)	9 (64.3)	10 (52.6)	11 (40.7)	9 (25.7)	18 (69.2)	17 (54.8)
Systemic therapy	2 (6.1)	6 (15.8)	2 (14.3)	8 (42.1)	7 (25.9)	4 (11.4)	7 (26.9)	13 (41.9)
Surgery contraindicated, *n* (%)	14 (42.4)	29 (76.3)	NA	NA	18 (66.7)	17 (48.6)	NA	NA

BCCNS = basal cell carcinoma nevus syndrome; EAS = expanded access study; ECOG PS = Eastern Cooperative Oncology Group performance status; laBCC = locally advanced basal cell carcinoma; mBCC = metastatic basal cell carcinoma; NA = not applicable.

### Treatment exposure

Because of early termination of the EAS study, the median duration of treatment with vismodegib was shorter in the EAS study than in the ERIVANCE BCC study. Median treatment durations in patients with aBCC aged ≥65 years and <65 years were 9.2 and 10.2 months in ERIVANCE BCC, respectively, and 5.5 and 5.4 months in the EAS, respectively. Information on cumulative exposure to study treatment is presented in Table 2.

**Table 2:** Exposure to study treatment

	ERIVANCE BCC (*N* = 104)				EAS (*N* = 119)			
	laBCC		mBCC		laBCC		mBCC	
	≥65 years (*n* = 33)	<65 years (*n* = 38)	≥65 years (*n* = 14)	<65 years (*n* = 19)	≥65 years (*n* = 27)	<65 years (*n* = 35)	≥65 years (*n* = 26)	<65 years (*n* = 31)
Median total No. of 150-mg capsules taken, *n* (range)	275 (25–564)	300 (118–555)	276 (19–378)	335 (56–500)	218 (32–530)	143 (41–553)	142 (32–581)	167 (13–585)
Median total cumulative dose (range), g	41 (4–85)	45 (18–83)	41 (3–57)	50 (8–75)	33 (5–80)	21 (6–83)	21 (5–87)	25 (2–88)

BCC = basal cell carcinoma; EAS = expanded access study; laBCC = locally advanced basal cell carcinoma; mBCC = metastatic basal cell carcinoma; NA = not applicable.

### Best overall response rate

**Figure 1:**
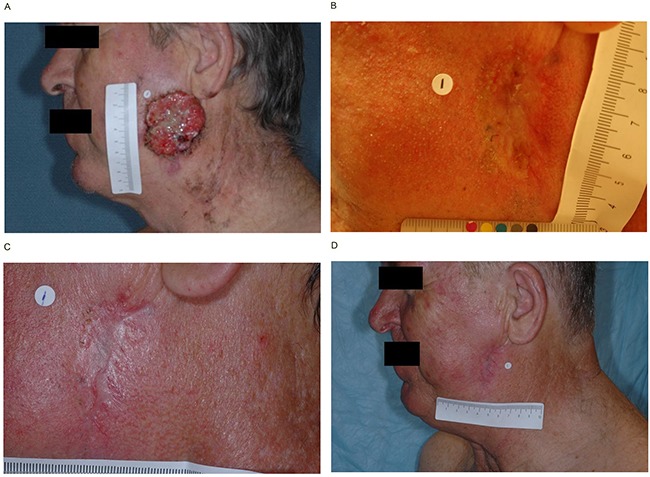
Representative examples of individual patient responses Patient 1: Single target lesion in a 59-year-old man at screening **A.** 8 weeks **B.** 16 weeks **C.** and 24 weeks **D.** Patient 2: Target lesion on the left temple in an 82-year-old man at screening **E.** 8 weeks **F.** 16 weeks **G.** and 24 weeks **H.**

Clinical activity was observed across all cohorts in both studies. In the ERIVANCE BCC study, the investigator-assessed best overall response rate (BORR) was 46.7% (95% confidence interval [CI] 28.3–65.7%) and 72.7% (54.5–86.7%) in patients with laBCC aged ≥65 and <65 years, respectively. In the EAS, the BORR was 45.8% (25.6–67.2%) and 46.9% (29.1–65.3%) in patients with laBCC aged ≥65 and <65 years, respectively. Among patients with mBCC, the BORR was 35.7% (95% CI 12.8–64.9%) and 52.6% (95% CI 28.9–75.6%) in patients aged ≥65 and <65 years, respectively, in the ERIVANCE BCC study, and 33.3% (95% CI 13.3–59%) and 28.6% (95% CI 11.3–52.2%) in patients aged ≥65 and <65 years, respectively, in the EAS (Table 3). Representative examples of individual patient responses are shown in Figure 1.

**Table 3:** Investigator-assessed best overall response (efficacy-evaluable patients)

	ERIVANCE BCC (*N* = 96)				EAS (*N* = 95)			
	laBCC		mBCC		laBCC		mBCC	
	≥65 years (*n* = 30)	<65 years (*n* = 33)	≥65 years (*n* = 14)	<65 years (*n* = 19)	≥65 years (*n* = 24)	<65 years (*n* = 32)	≥65 years (*n* = 18)	<65 years (*n* = 21)
BORR, *n* (%)	14 (46.7)	24 (72.7)	5 (35.7)	10 (52.6)	11 (45.8)	15 (46.9)	6 (33.3)	6 (28.6)
[95% CI]	[28.3–65.7]	[54.5–86.7]	[12.8–64.9]	[28.9–75.6]	[25.6–67.2]	[29.1–65.3]	[13.3–59]	[11.3–52.2]
Complete response	8 (26)	12 (36)	0	0	2 (8)	4 (12)	1 (6)	1 (5)
Partial response	6 (20)	12 (36)	5 (36)	10 (53)	9 (38)	11 (34)	5 (28)	5 (24)
Stable disease	11 (37)	4 (12)	7 (50)	8 (42)	12 (50)	15 (47)	9 (50)	11 (52)
Progressive disease	2 (7)	4 (12)	1 (7)	1 (5)	0	0	1 (6)	2 (10)
Not evaluable/missing	3 (10)	1 (3)	1 (7)	0	1 (4)	2 (6)	2 (11)	2 (10)

BCCNS = basal cell carcinoma nevus syndrome; BORR = best overall response rate; CI = confidence interval; EAS = expanded access study; laBCC = locally advanced basal cell carcinoma; mBCC = metastatic basal cell carcinoma.

### Safety

Within each trial, vismodegib demonstrated a safety profile in patients aged ≥65 years similar to that observed in younger patients (Table 4). The most frequent AEs in patients aged ≥65 *vs.* <65 years in ERIVANCE BCC and the EAS were muscle spasms (64% *vs.* 72% and 70% *vs.* 73%, respectively), dysgeusia (51% *vs.* 51% and 70% *vs.* 73%, respectively), and alopecia (49% *vs.* 75% and 55% *vs.* 61%, respectively). Grade 3-5 AEs in patients aged ≥65 *vs.* <65 years occurred in 51% *vs.* 35% of patients in ERIVANCE BCC and 25% *vs.* 21% of patients in EAS, respectively. Similarly, AEs leading to treatment discontinuation occurred in 15% *vs.* 11% and 11% *vs.* 2% of patients aged ≥65 *vs.* <65 years in ERIVANCE BCC and EAS, respectively. Although numerical differences in the incidence of AEs were observed across cohorts, no consistent trends were observed across studies. No new safety signals were identified.

**Table 4:** Selected adverse events

Selected AEs, n (%)	ERIVANCE BCC (*N* = 104)		EAS (*N* = 119)	
	≥65 years (*n* = 47)	<65 years (*n* = 57)	≥65 years (*n* = 53)	<65 years (*n* = 66)
Any AE	47 (100)	57 (100)	52 (98)	64 (97)
Grade 3-5 AE	24 (51)	20 (35)	13 (25)	14 (21)
AE leading to discontinuation	7 (15)	6 (11)	6 (11)	1 (2)
Muscle spasms	30 (64)	41 (72)	37 (70)	47 (73)
Dysgeusia	24 (51)	29 (51)	37 (70)	47 (73)
Alopecia	23 (49)	43 (75)	29 (55)	40 (61)
Nausea	12 (25)	18 (32)	9 (17)	14 (21)
Diarrhea	8 (17)	15 (26)	14 (26)	16 (24)
Ageusia	7 (15)	5 (9)	2 (4)	1 (2)
Constipation	11 (23)	6 (11)	5 (9)	9 (14)
Arthralgia	6 (13)	10 (18)	0	4 (6)
Decreased weight	23 (49)	25 (44)	10 (19)	9 (14)
Decreased appetite	14 (30)	10 (18)	7 (13)	9 (14)
Fatigue	17 (36)	20 (35)	10 (19)	13 (20)

AE = adverse event; BCCNS = basal cell carcinoma nevus syndrome; EAS = expanded access study.

## DISCUSSION

This analysis of the ERIVANCE BCC study and the EAS evaluated the efficacy and safety of vismodegib in patients with aBCC aged ≥65 years compared with those aged <65 years. Vismodegib demonstrated similar clinical activity across all patient cohorts, including patients aged ≥65 years. Numerical differences in response rates between cohorts were not considered to be clinically meaningful and were likely due to differences in response criteria, assessment schedule, treatment duration, and length of follow-up in each study.

Patients aged ≥65 years are often considered at greater risk of AEs because of the presence of comorbidities and age-related impairment of organ function [7]. Importantly, the incidence of common chronic conditions in these studies (ERIVANCE BCC and EAS) was generally similar to that in the general population (US Medicare fee-for-service beneficiaries) in both patients aged ≥65 years and younger patients, except for a lower incidence of diabetes (8–9% and 4–6% in patients aged ≥65 and <65 years, respectively, compared with a prevalence of 27% and 25%, respectively) [9]. In this analysis, the safety profile of vismodegib in patients aged ≥65 years appeared to be similar to that observed in younger patients. While numerical differences were observed between analytical cohorts, no consistent trends were observed across the 2 trials. These conditions often require treatment with medications known to influence hepatic drug metabolism when co-administered with inhibitors of drug-metabolizing and transporter enzymes [10,11]. However, the therapeutic index of vismodegib is broad, with no obvious relationship between exposure and AEs. Furthermore, results of clinical studies show that various cytochrome (CYP2C8, CYP3A4, CYP2C9) substrates and a proton pump inhibitor (rabeprazole) do not meaningfully alter the pharmacokinetic profile of vismodegib [10,11]. The similar AE profile and the tumor response in the <65- and ≥65-year-old patient cohorts treated with vismodegib make it reasonable to conclude that vismodegib is safe and effective and may be concomitantly administered with common medications used in patients aged ≥65 years with locally advanced or metastatic BCC.

## CONCLUSION

The results of this analysis suggest that patients aged ≥65 years are likely to experience benefit from vismodegib similar to that for younger patients without any apparent increase in the risk of AEs.

## MATERIALS AND METHODS

### Study design

ERIVANCE BCC (ClinicalTrials.gov number NCT00833417) was an international, multicenter, non-comparative phase 2 study. EAS (SHH4811g, ClinicalTrials.gov number NCT01160250) was a multicenter, open-label, noncomparative expanded access study to provide patients with aBCC who lack satisfactory treatment options access to vismodegib prior to regulatory approval. The EAS was terminated early because of FDA approval of vismodegib. All patients signed written informed consent before enrolling in either study.

### Key eligibility criteria

Key eligibility criteria for the ERIVANCE BCC and EAS studies were similar. Patients with mBCC were required to have histologic confirmation of distant metastasis. Patients with laBCC were required to have ≥1 lesion measuring ≥10 mm, inoperable or surgery contraindicated (e.g., recurrence after ≥2 prior surgeries and curative resection deemed unlikely, or anticipated substantial morbidity and/or deformity from surgery), and prior radiation to ≥1 lesion, unless contraindicated or inappropriate. Other criteria included age ≥18 years, adequate organ function, and Eastern Cooperative Oncology Group performance status ≤2. While the ERIVANCE BCC trial required patients to have measurable disease according to Response Evaluation Criteria in Solid Tumors, version 1.0 (RECIST v1.0) guidelines, the EAS study also allowed enrollment of patients with nonmeasurable disease. Both trials allowed enrollment of patients with BCCNS, as long as all other eligibility criteria were met.

### Treatment

In both studies, all patients received oral vismodegib 150 mg/day until disease progression, intolerable toxicity, patient withdrawal, or study termination.

### Assessments

#### Efficacy assessments

For mBCC, response was evaluated according to RECIST v1.0 in both ERIVANCE BCC and EAS. For laBCC, response was assessed according to RECIST v1.0 in the EAS and to a novel composite endpoint (≥30% reduction in externally visible or radiographic dimensions, or complete resolution of ulceration if present at baseline) in ERIVANCE BCC. Response assessments were performed every 8 weeks in ERIVANCE BCC and every 8–16 weeks in the EAS.

#### Safety assessments

Adverse events were assessed on a monthly basis in both trials and graded according to National Cancer Institute Common Terminology Criteria for Adverse events (NCI-CTCAE), version 3.0 in the ERIVANCE trial and NCI-CTCAE version 4.0 in the EAS trial.

### Analysis

All patient data available as of November 26, 2010, for ERIVANCE BCC (primary analysis) and April 23, 2012, for US EAS (final analysis) were included in the analyses. Analytic cohorts for patients aged ≥65 and <65 years were created within each trial for comparison using descriptive statistical methods. Data were not pooled across the trials because of the described differences in the schedule and the criteria for assessment of response. Best overall response rate was analyzed in efficacy-evaluable patients, and 95% CI was computed using the Clopper-Pearson method.
